# Diagnosis of Rosai-Dorfman Disease in Elderly Female on Fine Needle Aspiration Cytology: A Case Report

**DOI:** 10.1155/2012/806130

**Published:** 2012-10-14

**Authors:** Meher Aziz, Prasenjit Sen Ray, Nazima Haider, Sumit Prakash Rathore

**Affiliations:** Department of Pathology, J.N. Medical College, AMU, Aligarh 202002, India

## Abstract

Rosai-Dorfman disease (RDD) is a rare benign disorder of histiocytic proliferation that usually presents with bilateral cervical lymphadenopathy in children. We describe the case of a 50-year-old lady suffering from this disease who presented with generalized lymphadenopathy and a left sided chest wall lump. Fine needle aspiration cytology (FNAC) from all the lesions showed abundant benign histiocytes with lymphophagocytosis which was compatible with the diagnosis of RDD. This case is being reported for its rarity in presentation in an elderly female with both generalized nodal as well as extranodal manifestations.

## 1. Introduction

Rosai-Dorfman disease, also known as sinus histiocytosis with massive lymphadenopathy (SHML), is a rare benign disorder of histiocytic proliferation of unknown etiology. Although the disease has a predilection to affect cervical lymph nodes in adolescent children, cases with extranodal manifestations and involving all age groups have been reported [1].

## 2. Case Report

A 50-year-old Indian female presented with complaints of low grade intermittent fever off and on, weakness, and slowly enlarging painless nodules in the right side of her neck and right groin for the last one and half years. There was no history of night sweats, reduced appetite, or weight loss. Past medical history and drug history were also insignificant.

**Figure 1:**
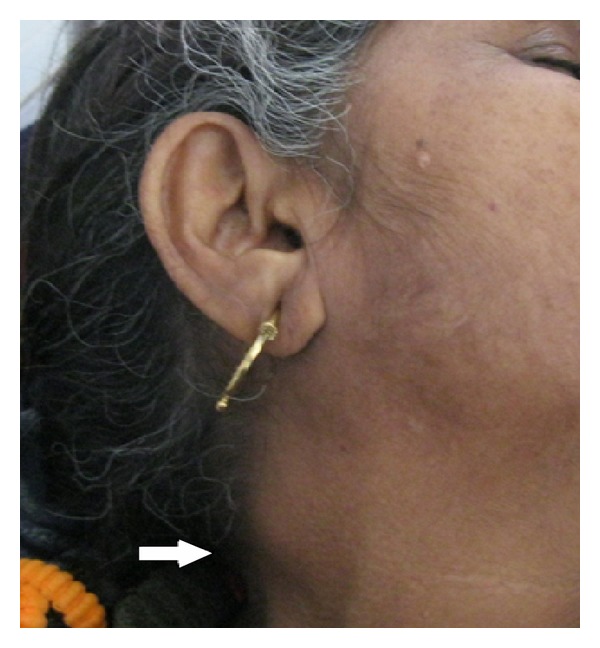
Enlarged right cervical lymph node, 2 × 1.5 cm.

Clinical examination revealed average built, mild pallor along with generalized lymphadenopathy involving right cervical (2 × 1.5 cm, multiple, matted) (Figure 1), right axillary (1 × 1 cm), and right inguinal (2 × 2.5 cm) lymph nodes. All the lymph nodes were nontender and firm in consistency. Another ill defined, non tender, firm lump (1 × 1 cm) was palpated over left side of her chest wall around 4th to 6th ribs in mid axillary line. On abdominal palpation, no hepato-splenomegaly was present. A provisional diagnosis of fever with generalized lymphadenopathy was made and the patient was admitted for further evaluation.

**Figure 2:**
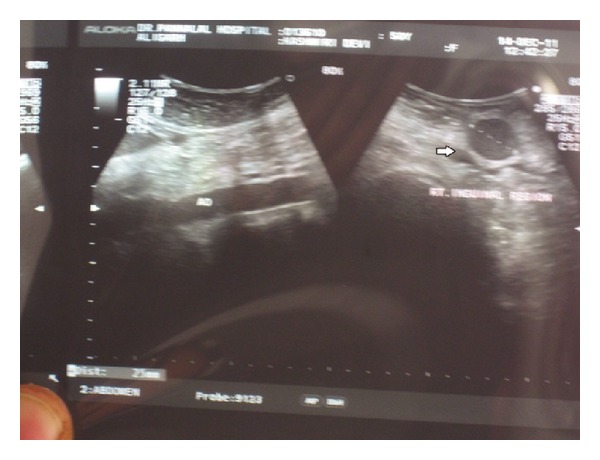
Ultrasound of enlarged right inguinal lymph node, 2.5 × 2 cm.

On routine haemogram, her haemoglobin was found to be 10.5 gm/dL, total leucocyte count 11,500/mm^3^, differential count N81 L16 E02 M01 B0, and platelet count 2,58,000/mm^3^. Peripheral smear examination showed normocytic to microcytic RBCs with mild hypochromia. No immature cells were present in the peripheral blood. Erythrocyte sedimentation rate (ESR) was 65 mm/hour. On chest X-ray, clear lung fields with enlarged hilar shadow were present. USG abdomen showed that liver and spleen were normal in shape, size, and echotexture. No free fluid or retroperitoneal lymphadenopathy was detected (Figure 2).

**Figure 3:**
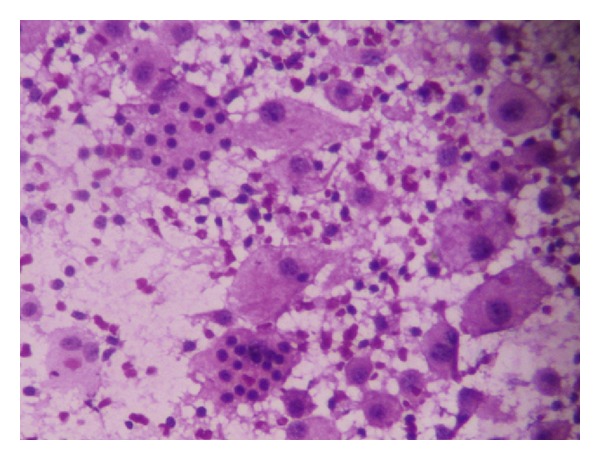
Many large histiocytes with binucleation and emperipolesis, (FNAC, H&E ×400).

Fine needle aspiration cytology (FNAC) from the enlarged lymph nodes as well as the chest wall lump revealed inflammatory infiltrate consisting of lymphocytes, plasma cells, sparse population of neutrophils along with multiple large histiocytes with abundant eosinophilic cytoplasm and vesicular nuclei, some showing binucleation. Many of these histiocytes showed emperipolesis (i.e., engulfment of intact lymphocytes and plasma cells). No malignant cells or granuloma were seen (Figure 3). These findings were consistent with the diagnosis of Rosai-Dorfman disease. The patient was finally diagnosed to be suffering from RDD with nodal and extranodal involvement.

## 3. Discussion

RDD was first described by Rosai and Dorfman in 1969 as sinus histiocytosis with massive lymphadenopathy [2]. It is a rare self-limiting benign disease of unknown etiology that is more prevalent among African Negros and has a predilection for males (male : female = 2 : 1). Although any age group can be affected, 80% of the cases manifest within the first two decades of life [3]. Classically, it presents with gradual onset massive bilateral painless cervical lymphadenopathy, fever, raised ESR, and hypergammaglobulinemia. Rosai and Dorfman [1] observed leucocytosis with neutrophilia in 19 out of the 34 cases of RDD in their study. Our patient was 50 years of age and had intermittent fever, leucocytosis, neutrophilia, and raised ESR. 

Involvement of axillary, inguinal, paraaortic, and mediastinal lymph nodes have also been documented in RDD [1]. Extranodal manifestation can be seen in 40–45% patients and tend to involve skin and subcutaneous tissue, salivary glands, orbit, respiratory tract, central nervous system, breast, bone marrow, and kidneys [3]. In our patient, nodal involvement was present in right cervical, axillary, and inguinal lymph nodes while extranodal involvement was confined to the skin of left chest wall. 

FNAC plays a useful role in the diagnosis of RDD. Aspirates from the affected lesions show proliferation of histiocytes with abundant eosinophilic cytoplasm, vesicular nuclei, and lymphophagocytosis or emperipolesis. In the latter, intact lymphocytes, plasma cells, and RBCs are found to be engulfed by the histiocytes and are a hallmark of RDD. In presence of these classical features on FNAC, a diagnosis of RDD can be reliably made, and as such, biopsy may be avoided [4, 5].

The differential diagnosis of RDD includes lymphoma, malignant histiocytosis, disseminated tuberculosis, and Langerhans cell histiocytosis (LCH) [6, 7]. The phenomenon of emperipolesis is central in differentiating RDD as the rest of these diseases fail to exhibit lymphophagocytosis. Presence of weight loss, night sweats, hepatosplenomegaly and malignant cells staining positive for CD45 favours the diagnosis of lymphoma. Malignant histiocytosis differs from RDD clinically by its rapid downhill course and pathologically by the presence of malignant histiocytes having bizarre, pleomorphic nuclei. The histiocytes in LCH have a characteristic folded and grooved nucleus and exhibit CD1a positivity. Disseminated tuberculosis can be ruled out on the basis of absence of granulomas and negative staining for acid fast bacilli by Ziehl-Neelsen stain [1, 6, 7].

In majority of the cases, RDD runs a benign self-limiting course and no treatment is necessary. However, in patients with massive nodal or extranodal involvement with threatening organ dysfunction, therapy is indicated. Although no precise treatment is known for this condition, multiple modalities including radiation, chemotherapy, glucocorticoids, interferon, and surgery have been attempted with variable outcome [8]. Our patient was put on oral Prednisolone and showed a decrease in the size of lymph nodes as well as abatement of fever. The patient is currently under regular followup.

## 4. Conclusion

Rosai-Dorfman disease is a rare condition which has both nodal and extranodal presentations and can often mimic a plethora of malignant neoplasms. However, given its benign and self-limiting course, the entity should be kept in mind so that unnecessary interventions to the patients can be avoided.
